# *SF3B1* Mutations Are Associated with Resistance to Non-Genotoxic MDM2 Inhibition in Chronic Lymphocytic Leukemia

**DOI:** 10.3390/ijms241411335

**Published:** 2023-07-12

**Authors:** Erhan Aptullahoglu, Jonathan P. Wallis, Helen Marr, Scott Marshall, Nick Bown, Elaine Willmore, John Lunec

**Affiliations:** 1 Medical Faculty, Newcastle University Cancer Centre, Newcastle upon Tyne NE2 4AD, UK; erhan.aptullahoglu@bilecik.edu.tr (E.A.); elaine.willmore@ncl.ac.uk (E.W.); 2 Department of Haematology, Freeman Hospital, Newcastle upon Tyne NHS Foundation Trust, Newcastle upon Tyne NE7 7DN, UK; jpwallis@btinternet.com (J.P.W.); helen.marr@nuth.nhs.uk (H.M.); 3 Department of Haematology, City Hospitals Sunderland NHS Trust, Sunderland SR4 7TP, UK; scott.marshall@chsft.nhs.uk; 4 Northern Genetics Service, Institute of Genetic Medicine, Newcastle upon Tyne NE1 4EP, UK; nick_bown@yahoo.co.uk

**Keywords:** *SF3B1*, splicing factor, MDM2-p53 antagonists, RG7388 (idasanutlin), chronic lymphocytic leukemia (CLL)

## Abstract

Chronic lymphocytic leukemia (CLL) is a genetically and clinically heterogeneous malignancy affecting older individuals. There are a number of current treatment options for CLL, including monoclonal antibodies, targeted drugs, chemotherapy, and different combinations of these. However, for those patients who are intrinsically treatment resistant, or relapse following initial responses, novel targeted therapies are still needed. Targeting the mouse double-minute-2 human homolog (MDM2), a primary negative regulator of p53, is an appealing therapeutic strategy for non-genotoxic reactivation of p53, since the *TP53* gene is in its wild-type state at diagnosis in approximately 90% of patients. Mutated *SF3B1* and *TP53* are both associated with more aggressive disease, resistance to therapies and poorer overall survival for CLL. In this study, we performed a screen for *SF3B1* and *TP53* mutations and tested RG7388 (idasanutlin), a second-generation MDM2 inhibitor, in a cohort of CLL primary patient samples. *SF3B1* mutations were detected in 24 of 195 cases (12.3%) and found associated with poor overall survival (hazard ratio [HR] 2.12, *p* = 0.032) and high CD38 expression (median CD38 (%) 32 vs. 5; *p* = 0.0087). The novel striking finding of this study was an independent link between *SF3B1* mutational status and poor response to RG7388. Overall, *SF3B1* mutations in CLL patient samples were associated with resistance to treatment with RG7388 ex vivo, and patients with the wild type for both *SF3B1* and *TP53* are more likely to benefit from treatment with MDM2 inhibitors.

## 1. Introduction

Chronic lymphocytic leukemia (CLL) is the most frequent type of leukemia affecting older individuals. The clinical course of CLL varies greatly from patient to patient [1]. Certain genomic alterations that disrupt the regulation of clonal B-cell proliferation and apoptosis are the starting event for leukemic transformation [2]. When treatment is needed, there are a number of therapeutic options currently available, including monotherapy with Bruton tyrosine kinase (BTK) inhibitors such as ibrutinib [3] and acalabrutinib [4], combination therapy with the B-cell lymphoma 2 (BCL2) inhibitor venetoclax with obinutuzumab [5], and chemoimmunotherapy [6]. Nonetheless, therapeutic resistance is common and thus there is still a need for novel targeted therapies that can cure distinct subgroups of patients with either intrinsic or acquired resistance to current treatment options.

Overall, approximately half of human cancers have mutations and/or deletions resulting in functional loss of the *TP53* gene which encodes the p53 tumor suppressor protein, highlighting the critical role of p53 in tumor suppression [7]. In contrast to many solid tumors, only approximately 10% of CLL cases are *TP53* deleted and/or mutated at diagnosis [8]. Targeting mouse double-minute-2 human homolog (MDM2) is an attractive therapeutic strategy for non-genotoxic reactivation of p53, since it serves as the key negative regulator of functional p53. One of the earliest studies with small-molecule MDM2 inhibitors in CLL showed that Nutlin-3a induced p53-mediated apoptosis in a majority of primary samples from untreated patients; all resistant samples had *TP53* mutations [9]. More potent and bioavailable compounds have been developed following the initial discovery of Nutlin-3a. RG7388 (idasanutlin) and HDM201 (siremadlin) are two prominent agents that are the subject of various pre-clinical and clinical trial studies. RG7388 activates wild-type p53, as evidenced by the stabilization of p53 and consequent increased expression of p53 transcriptional targets, including *MDM2*, *CDKN1A*(p21) and pro-apoptotic genes, such as *BBC3*(PUMA), *BAX* and *PMAIP1*(NOXA). This results in the inhibition of proliferation and induction of p53-dependent apoptosis in various cell types [10,11,12], including primary CLL cells [13]. RG7388 was the first MDM2-p53 binding antagonist to advance through phase II clinical trials (ClinicalTrials.gov Identifier: NCT02633059). Early clinical trial data indicate that RG7388 is a promising agent for use in anti-leukemic therapy strategies [14].

Splicing factor 3B subunit 1 (SF3B1) protein, encoded by the *SF3B1* gene, plays an important role in the recognition of the branchpoint sequence (BPS) and splicing of pre-mRNA that involves the linking of exons and the removal of introns to form mature mRNA [15,16]. *SF3B1* mutations have been associated with a number of splicing defects and have been identified in several cancer types [17,18] including CLL [19,20]. Among the other spliceosome mutations, the *SF3B1* mutations are the most prevalent and critical in hematological malignancies [21,22]. Mutated *SF3B1* is associated with more aggressive disease [23] and poorer overall survival in CLL [24]. The CLL8 trial has demonstrated that *SF3B1* and *TP53* mutations are among the strongest prognostic factors in CLL patients receiving chemoimmunotherapy [25]. The incidence of *SF3B1* mutations in newly diagnosed CLL patients and treated patients varies in the range from 5% to 17%, respectively [23,24]. These mutations are predominantly subclonal genetic events in CLL, corresponding to earlier events in CLL evolution [26]. Recent studies showed that *SF3B1* mutations induce RNA changes affecting multiple CLL-associated pathways including DNA damage response and Notch signaling [27]. *SF3B1* mutated CLL samples without concurrent *ATM* and *TP53* aberrations show defective apoptotic responses following treatment with DNA-damaging agents including fludarabine, doxorubicin or IR due to decreased upregulation of several genes including those encoding p21, BAX and PUMA [28].

Although the majority of p53 functional CLL primary samples exhibit a strong cytotoxic response to MDM2 inhibitors, a small subset of *TP53*^WT^ CLL samples show an intermediate response or resistance to such treatments. Here, we hypothesized that *SF3B1* mutational status could provide a predictive biomarker for response to MDM2-p53 binding antagonists.

## 2. Results

### 2.1. Sanger Sequencing Detects SF3B1 Mutations in CLL Samples

**Figure 1:**
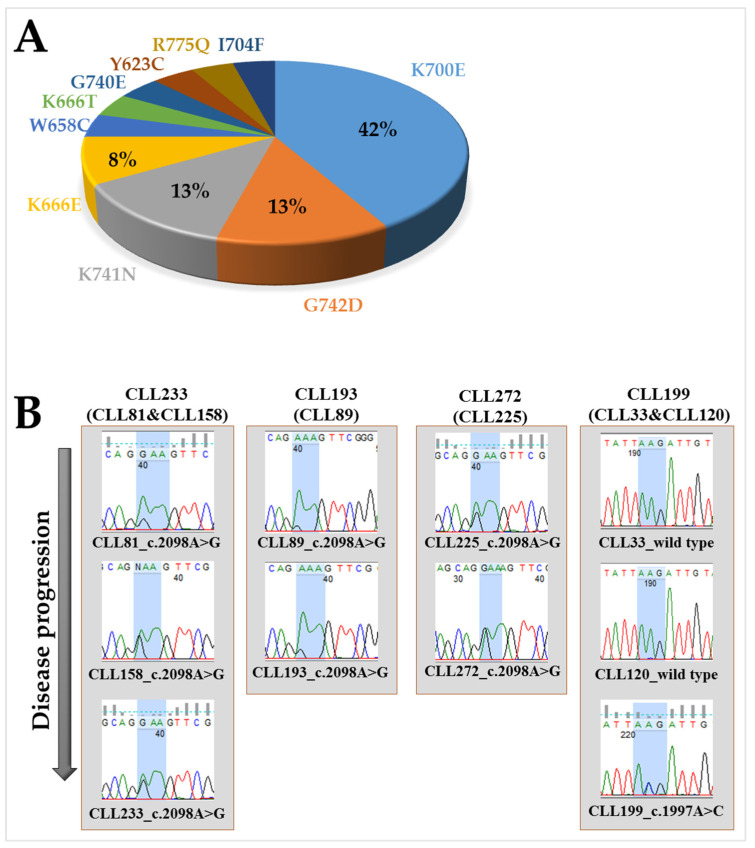
(**A**) The percentage distribution of the *SF3B1* mutations and respective amino acid positions across all CLL patient samples are displayed in a pie chart. (**B**) *SF3B1* mutations were seen with lower a allelic fraction in earlier samples taken from the same patient, indicating clonal expansion during leukemic progression. For the patient sample CLL199, the mutation in *SF3B1* (c.1997A>C; p.K666T) was not detected in the earlier samples CLL33 and CLL120.

Sequence analysis of CLL samples was performed for 195 patients from the Newcastle CLL Biobank. The analysis was conducted on 241 samples overall because some of the patients had multiple samples taken at various times after initial diagnoses. The most frequently altered regions of the *SF3B1* gene, exons 14, 15, 16, and 18, were analyzed. Mutations were found in 12.3% of CLL patients (24 out of 195 patients) at different stages of the disease (Supplementary Table S1). Two distinct mutations were present in one of the patients (CLL169). In exon 18, no mutation was detected. The most frequent mutation was c.2098A>G (10/24, 42%) in exon 15, resulting in the amino acid change from lysine to glutamic acid at amino acid position 700 (p.K700E). Nine different codons were found to be recurrently mutated, including codons 700 and 742 in 10 and 3 patients, respectively (Figure 1A).

Analysis of the clonal and subclonal mutations in a large CLL series revealed that *SF3B1* mutations are typically detected as subclonal mutations, particularly in early-stage samples, and hence likely to be a later event in the progression of CLL [26]. In our study, three of the *SF3B1*^MUT^ patients had the same mutation (c.2098A>G; p.K700E) in the earlier samples with lower a allelic fraction, supporting subclonal expansion during disease progression (Figure 1B). For one of the *SF3B1*^MUT^ patient samples (CLL199), the mutation was not detected in earlier samples (Figure 1B). Table 1 summarizes some of the clinical and molecular characteristics of the CLL cohort used in these analyses.

### 2.2. SF3B1 Mutations in CLL Samples Predict Poor Overall Patient Survival

**Figure 2:**
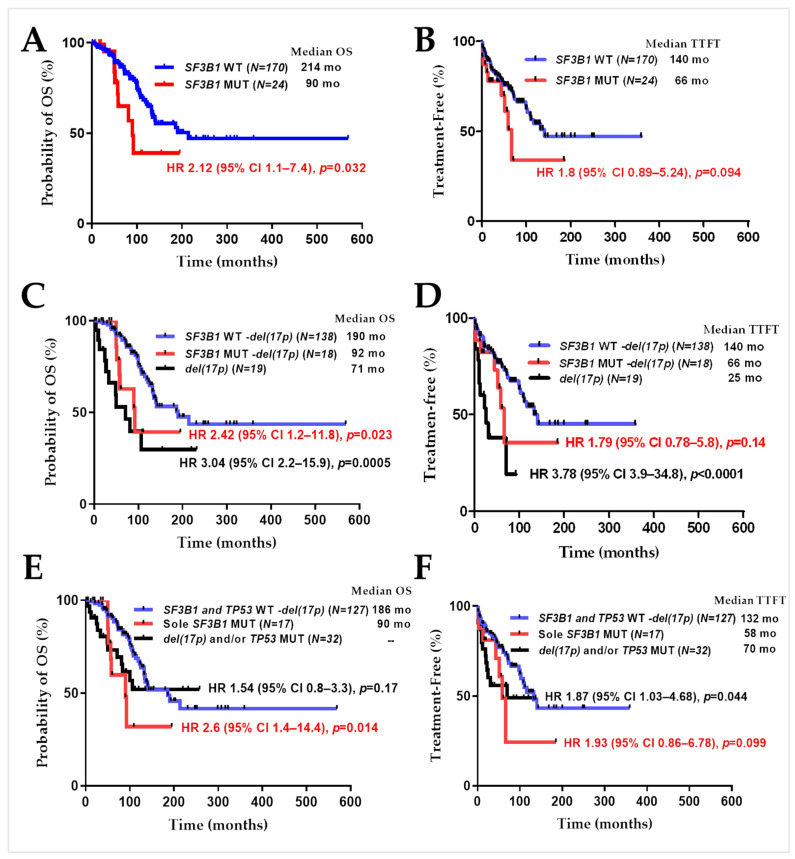
Survival plots of CLL patients depending on their *SF3B1* status. Kaplan–Meier curves of (**A**) overall survival (OS) and (**B**) treatment-free survival plot defined as time to first treatment (TTFT). Relationship of *SF3B1* mutations to (**C**) overall survival and (**D**) treatment-free survival by separating the subgroup of patients with del(17p) and/or *TP53* mutation. Blue line: *SF3B1*^WT^ without del(17p). Red line: *SF3B1*^MUT^ without del(17p). Black line: All cases with del(17p) independent of *SF3B1* status. (**E**) Overall survival and (**F**) treatment-free survival plot defined as time to first treatment. Blue line: Both *SF3B1* and *TP53* wild type (without del(17p)). Red line: *SF3B1*^MUT^ and *TP53*^WT^ (without del(17p)). Black line: All cases with del(17p) and/or *TP53* mutation independent of *SF3B1* status. The information next to the red and black lines shows the comparison with the blue lines in the corresponding graph. *p* values were calculated by log-rank statistics. HR: hazard ratio. CI: confidence interval.

The median follow-up was 190 months (range, 1–569 months) for 194 CLL patients. The presence of *SF3B1* mutations was significantly associated with lower overall survival (hazard ratio [HR] 2.12 (95% CI 1.1–7.4), log-rank test *p* = 0.032). The median overall survival was 90 months vs. 214 months for CLL cases with or without *SF3B1* mutations, respectively (Figure 2A). For all the CLL samples analyzed, the median time from diagnosis to first treatment was 132 months (range, 1–359 months). The median treatment-free survival was 66 months vs. 140 months for CLL cases with or without *SF3B1* mutations, respectively (hazard ratio [HR] 1.8 (95% CI 0.89–5.24), log-rank test *p* = 0.094) (Figure 2B).

Deletion of chromosome 17p distinguishes a subgroup of CLL patients with an exceptionally poor prognosis [29,30] and impaired response to MDM2 inhibitors [13]. The effect of *SF3B1* mutations on overall survival and treatment-free survival was also examined by separating the subgroup of patients with del17p. CLL patients with del17p (19 out of 175 patients) have a much lower survival rate than the patients without del17p (71 vs. 190 months, HR 3.04 (95% CI 2.2–15.9), log-rank test *p* = 0.0005) (Figure 2C). Patients carrying *SF3B1* mutations (-del17p) have shorter median overall survival compared those without *SF3B1* mutations and del17p (92 vs. 190 months, respectively; HR 2.42 (95% CI 1.2–11.8), log-rank test *p* = 0.023) and treatment-free survival (66 vs. 140 months, respectively; HR 1.79 (95% CI 0.78–5.8), log-rank test *p* = 0.14) (Figure 2C,D).

Mutations in *SF3B1* and *TP53* were not mutually exclusive (Table 1), as found in some patient cohorts in the literature [31,32]. To understand the sole effect of *SF3B1* mutations, we separated sole *SF3B1* mutated cases from patients displaying del17p and/or *TP53* mutations. Patients carrying sole *SF3B1* mutations (-del17p and *TP53*^WT^) have shorter median overall survival compared with those wild type for *SF3B1* and *TP53* genes (-del17p) (90 vs. 186 months, respectively; HR 2.6 (95% CI 1.4–14.4), log-rank test *p* = 0.014) and treatment-free survival (58 vs. 132 months, respectively; HR 1.93 (95% CI 0.86–6.78), log-rank test *p* = 0.099) (Figure 2E,F).

### 2.3. SF3B1 Mutations Were Associated with High CD38 Expression

**Figure 3:**
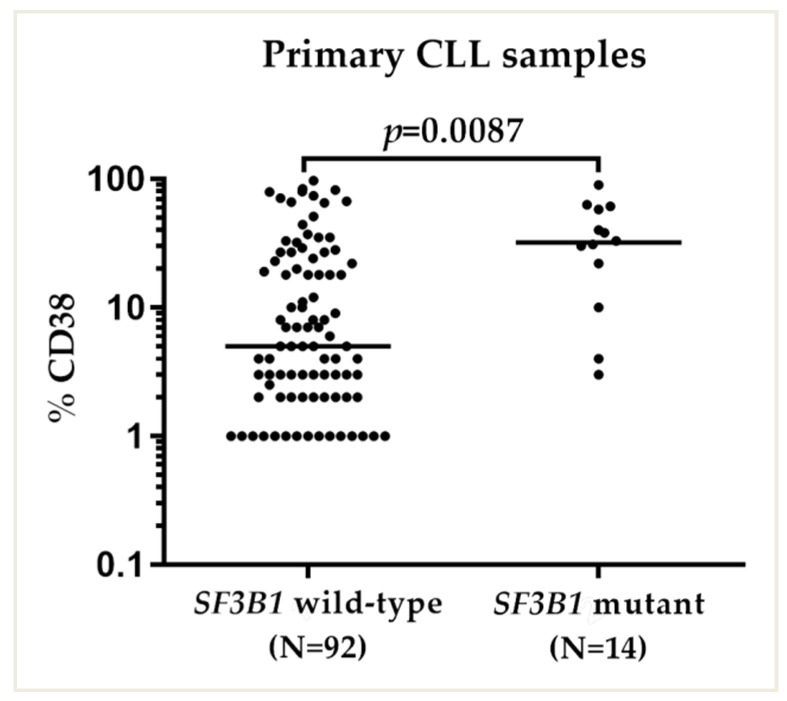
Primary CLL samples are grouped as *SF3B1*^MUT^ and *SF3B1*^WT^ according to their CD38 expression. CD38 status of primary CLL patient samples. Horizontal bars show the median CLL cell % positivity for CD38 expression. The % CD38 positivity was compared between *SF3B1* mutant and wild-type CLL samples. The *p* value shows a significant difference between the median values for the groups calculated using the Mann–Whitney *U* test.

A high percentage (>30%) of CLL cells in a patient sample positive for CD38 expression correlates with more aggressive clinical behavior [33]. Consistent with previous reports [34,35] in the current study a higher proportion of samples with *SF3B1* mutations were positive for CD38 (chi-square test, *p* = 0.0004) (Table 1). Overall, CD38 expression (in 30% or more of leukemic cells in a sample) was found in 25% (27 out of 110) of the patients. When the results of percentages of CD38-expressing cells were plotted individually, a higher proportion of CLL patients with mutated *SF3B1* had >30% CD38-positive tumor cells compared with those patients with wild-type *SF3B1* (median CD38 (%): 5 vs. 32; Mann–Whitney U test *p* = 0.0087) (Figure 3). The variable region of the immunoglobulin heavy chain (*IGVH*) gene is an established CLL prognostic biomarker. Advanced disease stages are closely associated with the absence of *IGVH* gene mutations [33]. Considering the classical *V_H_* homology cutoff value of <98% of alleles in a patient sample CLL cell population as *IGHV* mutated [36], 116 (59%) patients were classified as mutated and 79 (41%) patients as unmutated. *IGVH* status did not appear to correlate with *SF3B1* mutational status (chi-square test, *p* = 0.25) (Table 1).

### 2.4. SF3B1 Mutations Negatively Predict for Response to RG7388 in CLL Samples

**Figure 4:**
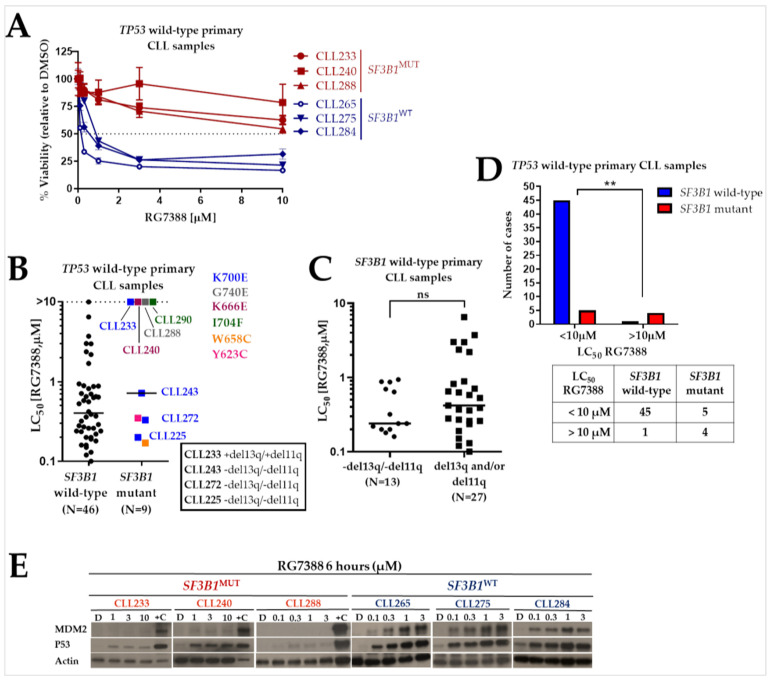
Comparison of response to RG7388 in CLL samples with respect to *SF3B1* mutational status. (**A**) Representative cytotoxicity curves for three *SF3B1*^MUT^ (CLL233, 240 and 288) and three *SF3B1*^WT^ (CLL265, 275 and 284) CLL samples exposed to increasing concentrations of RG7388 (from 0 to 10 µM) for 48 h. All six samples are wild type for the *TP53* gene. The *SF3B1*^MUT^ samples were highly resistant to RG7388 (LC_50_ > 10 µM). Cell viability was assessed by XTT assay. (**B**) LC_50_ values showing the range of responses to RG7388 in *TP53*^WT^ CLL samples. *SF3B1*^MUT^ samples are marked with different colors and the mutated codons are indicated beside the graph. CLL233 is the only one resistant to RG7388 among the four CLL samples with the identical *SF3B1*^K700E^ mutation (shown in blue). (**C**) LC_50_ values showing the range of responses to RG7388 in *SF3B1*^WT^ CLL samples. Horizontal bars show the median. There was no significant difference between the primary samples lacking both del13q and del11q, clinically relevant cytogenetic abnormalities in CLL, and samples carrying either or both of these abnormalities (unpaired *t*-test; *p* = 0.14). ns: not significant. (**D**) The majority of (4 out of 5) the highly resistant (LC_50_ > 10 µM) CLL samples were found to be *SF3B1*^MUT^. The chi-square test was used to measure whether the proportion of cases with LC_50_ values greater than 10 µM is statistically significant between the *SF3B1*^WT^ and *SF3B1*^MUT^ groups. **: *p* = 0.0017. The table below the bar graph shows the total number of CLL samples belonging to the related group. (**E**) Western blots of *SF3B1*^MUT^ CLL samples showed low levels of p53 increase saturated at the lowest drug concentration without activation of the downstream protein MDM2. On the other hand, concentration-dependent stabilization of p53 and the activation of the downstream protein MDM2 was evident in the *SF3B1*^WT^ samples. Actin was used as loading control. D: DMSO-treated control cell lysate; +C: positive control lysate from NGP cells treated with 2.5 µM Nutlin-3a for 4 h; MUT: mutant; WT: wild type.

Although *TP53*^WT^ primary CLL cells have a functional p53 pathway and are expected to be more sensitive to treatment with MDM2 inhibitors than samples with mutated/deleted *TP53*, a small subset shows an intermediate response or resistance to RG7388 (Figure 4A,B). To understand whether the expression of a mutated version of the *SF3B1* gene has an apparent impact on cellular response to the MDM2 inhibitors, the LC_50_ values of RG7388 for the p53 functional (*TP53*^WT^) CLL samples were plotted against the *SF3B1* status. It is noteworthy that 4 out of 5 samples that are highly resistant to RG7388 (LC_50_ > 10 µM) were *SF3B1* mutant (Figure 4B). The fraction of RG7388-resistant cases was considerably higher in the *SF3B1* mutant group compared to the *SF3B1* wild-type group (chi-square test, *p* = 0.0017) (Figure 4D).

To evaluate the functional integrity of the p53 pathway in the resistant samples with wild-type *TP53* (without del17p), a Western immunoblot was performed for three resistant (RG7388 LC_50_ > 10 µM) CLL samples with *SF3B1* mutation, and three sensitive (RG7388 LC_50_ = 0.57 ± 0.17 µM) CLL samples without *SF3B1* mutation (Figure 4A). Western blots of *SF3B1*^MUT^ CLL samples showed only low levels of p53 increase saturated at the lowest drug concentration without activation of the downstream protein MDM2. *SF3B1*^WT^ samples, on the other hand, show concentration-dependent stabilization of p53 and activation of the downstream protein MDM2 (Figure 4E).

In CLL primary samples treated with RG7388, the prognostic value of some of the critical biological and clinical indicators associated with the disease was investigated. The results were evaluated by univariate and multivariate analysis. Three distinct patient subgroups of responders to RG7388 (LC50 ≤ 1 µM: sensitive; 1 µM < LC50 < 10 µM: moderate; and LC50 ≥ 10 µM: resistant) were analyzed individually to better understand the roles played by particular variables in the varying response to the inhibitor. The parameters that are thought to be significant predictors of prognosis were used to build a multivariate comparisons model excluding *IGVH* and treatment status, CD38 expression and FISH stratification that had no or only borderline significance in the univariate analysis. *TP53* and *SF3B1* genes were found to be distinctive prognostic markers for the RG7388 treatment, supported in both univariate and multivariate analyses (Table 2).

### 2.5. Nalm-6 SF3B1^K700E^ Cells Are Less Sensitive to Growth Inhibition by RG7388 or HDM201 Compared to Matched SF3B1^K700K^

**Figure 5:**
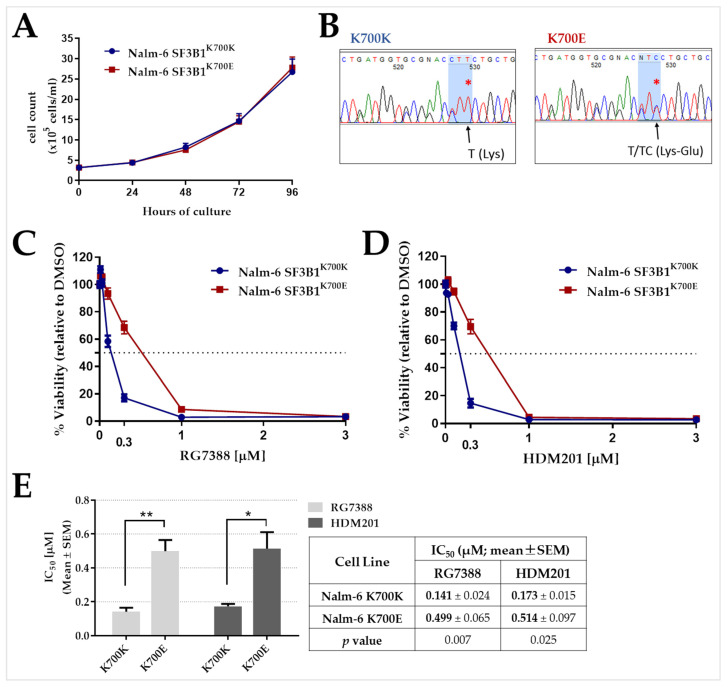
Comparative response of Nalm-6 isogenic cells expressing either SF3B1^K700K^ or SF3B1^K700E^ to different MDM2-p53 binding antagonists. (**A**) Growth curves of Nalm-6 isogenic cell lines over a period of four days in culture. The number of viable cells was assessed by trypan blue exclusion. Error bars show the mean ± standard error of the mean (SEM) of three independent counts of viable cells observed under trypan blue exclusion assay. (**B**) Sanger sequencing showing Nalm-6^K700E^ has a heterozygous point missense mutation c.2098A>G resulted in the amino acid change from lysine to glutamic acid at amino acid position 700 (K700E). Multiple silent mutations around the target point mutation were introduced to disrupt the PAM sequence and prevent re-cutting of the modified allele by Cas9. The sequencing was primed in the reverse direction of the coding strand. Red asterisks show the point mutation. (**C**,**D**) Pre-B isogenic Nalm-6 cells were treated with different concentrations of MDM2 inhibitors either RG7388 or HDM201 for 72 h. The % viability was measured by an XTT assay and normalized to DMSO solvent control treatment. Data are representative of three independent experiments and the error bars show the mean ± standard error of the mean (SEM) of at least three independent repeats. (**E**) Summary of IC_50_ (mean ± SEM) values for RG7388 and HDM201 in Nalm-6 isogenic cell lines from three independent repeats. Statistically significant differences between the cell lines (* *p* ˂ 0.05; ** *p* ˂ 0.01) are indicated.

A CRISPR engineered Nalm-6 isogenic cell line pair expressing either endogenous SF3B1^K700E^ or mutant SF3B1^K700K^ was used to further assess the effect of the most frequent *SF3B1* mutation, which has been reported to impair splicing in CLL [27]. Firstly, it was shown that both cell lines have comparable growth characteristics under the same conditions (Figure 5A). Sanger sequencing results confirmed the heterozygous point missense mutation at position c.2098A>G encoding the amino acid change from lysine to glutamic acid at amino acid position 700 (K700E) and the paired silent mutation control (K700K) (Figure 5B).

Cell line sensitivity to two different MDM2 inhibitors, RG7388 and HDM201 was tested. Nalm-6 cells expressing SF3B1^K700E^ were significantly less sensitive to growth inhibition following treatment with either RG7388 (unpaired *t* test, *p* = 0.007; Figure 5C,E) or HDM201 (unpaired *t* test, *p* = 0.025; Figure 5D,E) compared to the otherwise isogenic control cell line. 

### 2.6. SF3B1 Suppression Has No Effect on the Proliferation of B Cells and Their Response to MDM2 Inhibition

**Figure 6:**
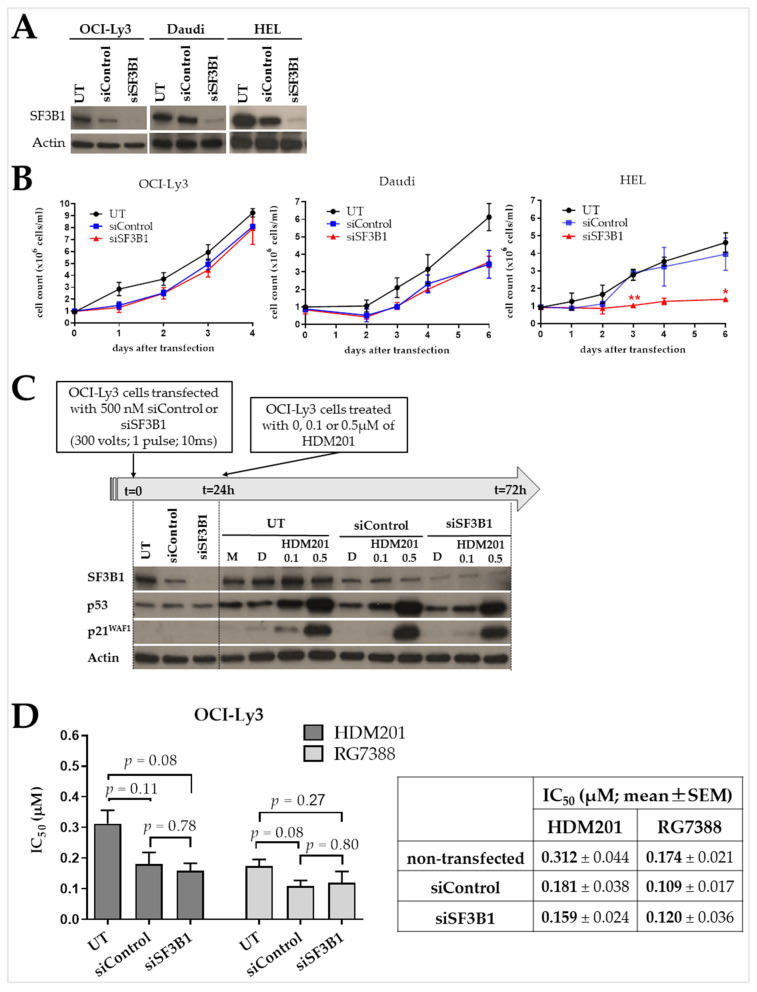
(**A**) Each cell line transfected with 500 nM siRNA targeting SF3B1 was compared with the corresponding cell line transfected with 500 nM scrambled siRNA control. SF3B1 protein levels measured 48 h post-transfection. Actin was used as loading control. (**B**) Cell count of viable cells for three cell lines with SF3B1 knockdown, compared with untreated and control siRNA transfected cells, as assessed by trypan blue exclusion assay. There was no statistically significant difference between the growth of siControl and siSF3B1 for OCI-Ly3 and Daudi cells (unpaired *t*-test; *p* > 0.05). Growth of HEL cells was inhibited with siSF3B1. Error bars show the mean ± SEM. Statistical analysis was performed by t-test and corrected for multiple comparisons using the Holm-Sidak method (* *p* < 0.05; ** *p* < 0.01 different from siControl control). (**C**) Schedule of treatment and immunoblot showing p53^WT^ OCI-Ly3 cells either non-transfected (UT) or transfected with 500 nM siControl or siSF3B1 24 h prior to treatment with 0, 0.1 or 0.5 μM of HDM201. HDM201 stabilized p53, with subsequent activation of downstream protein, p21^WAF1^. Actin was used as loading control. (**D**) Summary of IC_50_ values for HDM201 and RG7388 in OCI-Ly3 cells either non-transfected (UT) or transfected with 500 nM siControl or siSF3B1 24 h before treatment with the compounds. Viability measured by an XTT assay 48 h post-treatment with HDM201 or RG7388. There was no significant difference between the cells transfected with the siControl and siSF3B1 (*p* = 0.78 for HDM201; *p* = 0.80 for RG7388). Data are presented as the mean ± SEM for three independent repeats. M: only media control cell lysate; D: DMSO-treated control cell lysate. UT: untreated (non-transfected).

To evaluate the effect of a reduced level of SF3B1 on cell growth, three different cell lines including two B-cell lines (OCI-Ly3 and Daudi) and one myeloid cell line (HEL) were transfected with either siControl or siSF3B1. Cell proliferation after siRNA transfection was assessed by trypan blue exclusion assay and compared with the non-transfected control. Results showed that siRNA-mediated knockdown of SF3B1 had no significant effect on the proliferation of B-cell lines (OCI-Ly3 and Daudi) despite the high level of SF3B1 suppression. However, SF3B1 knockdown prevented HEL, a myeloid cell line, from proliferating (Figure 6A,B).

To understand the potential effect of SF3B1 knockdown on response to MDM2 inhibition, siRNA mediated SF3B1 knockdown was performed in the p53^WT^ B cell line, OCI-Ly3. OCI-Ly3 cells were treated with the MDM2 inhibitor, HDM201, 24 h after SF3B1 protein knockdown. For the analysis of protein levels and knockdown efficiency, cells were harvested 24 h after transfection, and also 48 h after treatment with two different concentrations of HDM201 (0.1 and 0.5 μM). Treatment with HDM201 stabilized p53, followed by activation of its transcriptional target p21^WAF1^ in OCI-Ly3. However, in contrast to the effect of SF3B1 mutation, there was no significant difference in the response patterns between control siRNA and siSF3B1 transfected cells despite the high level of SF3B1 suppression (Figure 6C). 50% growth inhibitory values (IC_50_s) for transfected and non-transfected cells were measured using an XTT cell viability assay 48 h after treatment with HDM201 or RG7388. Transfecting cells with siRNA resulted in a slight increase in sensitivity to HDM201 (not significant; unpaired *t*-test; UT vs. siControl *p* = 0.11; UT vs. siSF3B1 *p* = 0.08), but there was no significant difference between the cells transfected with the siControl and siSF3B1 (not significant; unpaired *t*-test *p*=0.78). The sensitivity of the cells was unaffected by SF3B1 suppression in RG7388-treated cells, either (not significant; siControl vs. siSF3B1 unpaired *t*-test *p* = 0.80) (Figure 6D).

## 3. Discussion

There are several splicing modulators that have been developed and reported to impair the normal functioning of the spliceosome complex and prevent cancer cell proliferation [37,38,39]. Some of these have a therapeutic potential in spliceosome-mutant cancers [40], while others inhibit splicing and cancer cell growth irrespective of splicing factor mutation status [41]. In our previous studies, we have proposed as a combined treatment strategy that spliceosome inhibition sensitizes B cell lines and CLL primary patient samples to non-genotoxic MDM2-p53 binding antagonists [42] which have previously been shown to be a promising single treatment option for CLL [13]. Splicing modulation by the spliceosome inhibitor E7107 resulted in the production of aberrantly spliced transcripts and protein products of p53 pathway genes, ultimately making B cells more susceptible to MDM2 inhibition [42].

Splicing factor mutations, with the *SF3B1* gene mutation being one of the most prominent of these, are another factor that interferes with the normal splicing function of cells and has been shown to play a significant role in carcinogenesis [43]. A defective spliceosome function induces aberrant endogenous splicing activity affecting multiple CLL-associated signaling pathways including the Notch [27] and DNA damage response (DDR) [44] pathways. Mutated *SF3B1* is associated with more aggressive disease [23], lower responses to chemoimmunotherapy, and poorer overall survival in CLL [24]. These results have led us to ask the question of whether *SF3B1*, as a critical component of the spliceosome complex and known to be frequently mutated in CLL, is a marker in the variable response and unexplained resistance to the MDM2 inhibitors in a small subset of patient samples. We first aimed to determine the *SF3B1* and *TP53* mutational status of our CLL cohort. In the clinically and genetically heterogeneous CLL cohort, *SF3B1* mutations were detected in 12.3% of cases. Three of the mutated cases had the same point mutation with lower a allelic fraction in the earlier samples, indicating subclonal expansion during progression. The maximum percentage of all sequencing peaks for the mutant allele was 50%, which is consistent with the fact that these somatic heterozygous point missense mutations behave in a genetically dominant fashion [17].

High levels (>30%) of CD38-positive cells in CLL samples have been associated with more aggressive clinical behavior [33,45]. In recent years, however, its contribution in guiding treatment with targeted agents has been found to be limited, and as a result, *TP53* and *IGHV* status have gained more prominence [46]. In the present study, *SF3B1* mutations were found more frequently in patient samples with CD38 expression in >30% of CLL cells. CLL patients with mutated *SF3B1* had significantly higher median values for the percentages of CD38-positive cells compared with those patients with wild-type *SF3B1* (Figure 3). This was a finding that supports the earlier literature on CD38 expression [33] and that *SF3B1* mutations are more prevalent in this subgroup of CLL patients with poor clinical behavior [23]. We then hypothesized that *SF3B1* is a prospective candidate that alone could drive the variable response to the MDM2 inhibitor RG7388.

Although MDM2 inhibitors exhibit a significant cytotoxic effect on the majority of *TP53*^WT^ CLL primary samples, an intermediate response or resistance to RG7388 treatment in a small subset of *TP53*^WT^ CLL samples (Figure 4B) emerged as an important finding that should be further investigated. In terms of potential mechanisms for this varied response, it is possible that some *TP53*^WT^ CLL samples may be less sensitive to MDM2 inhibitors due to impaired function of downstream targets of p53. To understand whether the mutated *SF3B1* expression has an apparent impact on response to the MDM2 inhibitors, the LC_50_ values of RG7388 for the p53 functional (*TP53*^WT^) CLL samples were plotted against the *SF3B1* status. The proportion of RG7388-resistant cases in the *SF3B1* mutant group was significantly different from that in the *SF3B1* wild-type group (Figure 4D), suggesting that the *SF3B1* mutations can modulate protein function in CLL in ways that alter drug response. This is consistent with evidence of a low level of p53 stabilization without activation of downstream protein MDM2 in *TP53*^WT^ (without del17p) but *SF3B1*^MUT^ CLL primary samples treated with increasing concentrations of RG7388 (Figure 4A,E). Several p53 pathway-related protein isoforms with failed function, including p53 itself, have been identified to exist in tumors [47,48,49,50]. In context of the available evidence in this study, it is probable that the *SF3B1* mutations impart an extensive alternative mRNA splicing profile, including aberrant isoforms and protein products of p53 pathway genes, which contributes to the mechanism of the resistance in B-cells. As a result, the main mechanism of action of MDM2 inhibitors—regaining the tumor suppression function of the p53 protein—is restricted.

These results, however, led to the question of how the samples with the identical c.2098A>G (p.K700E) point mutation respond to RG7388 differently. The reason for this is not clear, but obviously in addition to the *SF3B1* mutational status, additional factors such as known clinically relevant cytogenetic abnormalities, e.g., del11q or del13q, BCL2-family proteins, and/or other driving mutations may come into play. In contrast to the other three CLL samples carrying the identical mutation, we noticed that the RG7388-resistant sample with the p.K700E alteration (CLL233) carried both del13q and del11q chromosomal defects. In connection with this, a study in a murine model of CLL showed that heterozygous *Sf3b1* mutation and *Atm* defects (11q deletion and/or mutation) together can generate clonal expansions of mature B cells consistent with clinical pathologic features of CLL [51].

When we performed regression analyses with prognostic markers that could potentially be predictors of the response to RG7388 in CLL, *TP53* and *SF3B1* gene status were the only statistically significant factors. The mutation statuses of both genes were found to be statistically significant independent predictive variables for response to RG7388, supported in both univariate and multivariate analyses (Table 2). Interestingly, and supporting the interpretation in Figure 4A,B, *TP53* status specifically distinguished a subgroup of intermediate responders (1 µM < LC_50_ < 10 µM) from the sensitive subgroup (LC_50_ ≤ 1 µM), while *SF3B1* was a more prominent biomarker in separating resistant responders (LC_50_ ≥ 10 µM). We have previously shown that the *TP53* status in the subgroup of patients who are moderately responsive to RG7388 differs from the drug-sensitive group, and the *TP53* mutant allele frequency has also an effect on the variable response [13]. The frequency of *SF3B1* mutations, especially in the highly resistant samples (LC_50_ ≥ 10 µM), unlike *TP53*, can be explained by inducing RNA changes belonging to multiple CLL-associated pathways [27]. Furthermore, Nalm-6 isogenic cells with the most frequent heterozygous point missense *SF3B1* mutation (c.2098A>G; K700E) were tested for a causal link and found to be relatively more resistant to growth inhibition by RG7388 or HDM201 compared to matched isogenic control *SF3B1* wild-type cells (Figure 5). We successfully demonstrated that the introduction of the *SF3B1* mutation confers resistance to RG7388 in Nalm-6 isogenic cells. However, the observed effect remained limited compared to ex vivo assays, although there was a clear decrease in sensitivity compared to matched isogenic control. It should be noted that due to the limited availability of *TP53* wild-type CLL cell lines and difficulties in mutation introduction, the acute lymphoblastic leukemia cell line (but B cell precursor leukemia) was used here as a model.

To test whether silencing the *SF3B1* gene has similar effects as the gene mutations, siRNA-mediated knockdown was performed in three different cell lines including two B-cell lines (OCI-Ly3 and Daudi) and one myeloid cell line (HEL). SiRNA-mediated knockdown of SF3B1 did not have any effect on OCI-Ly3 and Daudi cell growth, despite a substantial reduction in the level of SF3B1 protein. OCI-Ly3 cells were also treated with a second-generation MDM2 inhibitor HDM201 after SF3B1 knockdown. There was no apparent difference between siControl and siSF3B1 transfected cells in terms of p53 stabilization and its target gene activation despite a high level of SF3B1 suppression. In addition, transfecting cells with siSF3B1 did not change the sensitivity to HDM201 and RG7388 compared to the cells transfected with siControl (Figure 6). These results support previous literature that the *SF3B1* mutations are dominant change-of-function mutations rather than loss-of-function mutations [52,53], although we established the effect of *SF3B1* mutations in a different cell line, Nalm-6, apart from the cell lines used in the knockdown experiments. Further research regarding the effects of SF3B1 knockdown in Nalm-6 cells would be worthwhile considering our findings indicate the *SF3B1* mutation reduces sensitivity to MDM2 inhibition. The impact of SF3B1 knockdown on growth was also evaluated on the myeloid cell line, HEL, as a control cell line. Interestingly, SF3B1 knockdown inhibited cell growth in this *SF3B1*^WT^ myeloid cell line. These results reflect the findings of Dolatshad et al., who reported growth inhibition and induction of cell cycle arrest as a result of SF3B1 suppression in myeloid cells [54], and also provide evidence that the myeloid and lymphoid lineages of blood cells behave in a different way in response to SF3B1 knockdown, as myeloid lineage cancer cells showed a particular vulnerability to the consequences of SF3B1 suppression. Investigating the reasons behind the difference may be useful for further understanding the role of *SF3B1* mutations in hematological malignancies.

Richter’s transformation (RT), also referred to as Richter’s syndrome, is the transformation of CLL or small lymphocytic lymphoma (SLL) into a new and more aggressive malignant disease [55]. Its pathophysiology has been extensively studied in terms of genetic and molecular alterations, and new research tools have made it simpler to diagnose, but there are still challenges with its management [56]. The reported prevalence of RT has not dropped in the era of new targeted medicines and keeps varying throughout clinical trials [57,58,59,60,61]. The majority of patients have chemoimmunotherapy resistance [62], and even novel therapies do not appear to significantly improve prognosis [63]. Last but not least, we would like to emphasize the value of further investigation into the response rates to RG7388 in groups of SF3B1 wild-type versus mutant patients with Richter syndrome.

## 4. Materials and Methods

### 4.1. Cell Lines and Compounds

OCI-Ly3, Daudi and HEL cell lines were obtained from authenticated cell line resources (ATCC or DSMZ) and routinely cultured using RPMI-1640 medium (Sigma-Aldrich, St. Louis, MO, USA) with 10% fetal calf serum and 100 U/mL penicillin/streptomycin (Sigma-Aldrich, St. Louis, MO, USA). Gene edited Nalm-6 isogenic cell lines expressing either endogenous SF3B1^K700K^ or mutant SF3B1^K700E^ were provided by H3 Biomedicine (Cambridge, MA, USA) and cultured as described above. Siremadlin (HDM201) and idasanutlin (RG7388) were dissolved in DMSO (Sigma-Aldrich, St. Louis, MO, USA) and used at a final concentration of 0.5% DMSO (*v*/*v*). Both compounds were custom synthesized with >99% purity as part of the Newcastle University/Astex Pharmaceuticals Alliance and CRUK Drug Discovery Programme at the Newcastle University Cancer Centre.

### 4.2. Patient Samples

Peripheral blood samples were obtained from CLL patients with informed consent, in accordance with institutional guidelines and the Declaration of Helsinki. CLL patient samples were obtained and stored under the auspices of the Newcastle Biobank (Research Ethics Committee (REC) reference 17/NE/0361). CLL diagnosis was made according to IWCLL-164 NCI 2008 criteria [64]. Peripheral blood samples were obtained from CLL patients with total white blood cell counts of at least 30 × 10^9^ cells/L, supporting the high proportion of malignant CD5+/CD19+ B cells. Mononuclear cells were purified by density gradient centrifugation (Lymphoprep, Axis-Shield Ltd., Dundee, UK) following the manufacturer’s protocol. Sensitivity to MDM2 inhibitors was always assessed on fresh samples just after collecting from the patients.

### 4.3. Patient Sample Information

The details of Sanger sequencing of *SF3B1* in primary CLL samples have been described in a previous study [42]. The *TP53* mutational status of CLL samples was assessed by next-generation sequencing (using Roche 454 GS FLX and Illumina MiSeq platforms) in all samples. The presence of a 17p deletion was assessed by fluorescence in situ hybridization and/or multiplex ligation-dependent probe amplification analysis. The functional status of p53 in CLL samples was determined by observing the stabilization of p53 and activation of downstream protein, p21^WAF1^, following short-term exposure to the MDM2 inhibitor RG7388.

CD38 status was measured as described previously [65] in cells that had been frozen (90% fetal bovine serum (Gibco, Life Technologies, Carlsbad, CA, USA) and 10% DMSO (Sigma-Aldrich, St. Louis, MO, USA) at the time of collection) and samples were classed as positive if the expression was >30%. Viability (routinely assessed by trypan blue exclusion assay) was >95% in fresh and thawed samples after 24 h culture. Binet staging refers to the patient status at the time of sample collection. *IGHV* analysis was carried out by Paul Evans (St James’s University Hospital, Leeds, UK) within the scope of a project previously implemented jointly with our group [66].

### 4.4. Cell Viability Assay

#### 4.4.1. Cell Lines

Pre-B isogenic Nalm-6 cells were seeded at 1.6 × 10^5^ cells/mL in 100 μL of medium per well of a 96-well plate (Corning) for 24 h before treating with a range of concentrations (from 1 to 10^4^ nM) of siremadlin or idasanutlin for 72 h. XTT Assay Kit II (Sigma-Aldrich, Gillingham, UK) was used to measure growth inhibition compared to solvent DMSO control.

For knockdown experiments, OCI-Ly3 cells either non-transfected or transfected with 500 nM siControl or siSF3B1 24 h before treatment with the compounds. Viability measured by an XTT assay 48 h post-treatment with siremadlin or idasanutlin.

#### 4.4.2. PBMCs

The 5 × 10^6^ cells/mL in 100 μL of medium per well of a 96-well were exposed to a range of concentrations (from 1 to 10^4^ nM) of idasanutlin for 48 h. Ex vivo cytotoxicity was assessed by XTT Assay Kit II (Sigma-Aldrich, Gillingham, UK). Results were normalized to DMSO controls and expressed as % viability.

### 4.5. Design of Synthetic siRNA Molecules

Three different SF3B1-targeting siRNA constructs and the control (scrambled siRNA or siControl) were designed and custom synthesized by Eurogentec Ltd. (Southampton, UK). The knockdown conditions were optimized using the following siRNAs (sequence 5′-3′), and siRNA#1 was used in the experiments (Supplementary Figure S1).

- siSF3B1#1 Sense: CUAGAGAAGUGAUGUUAAU, Antisense: AUUAACAUCACUUCUCUAG
- siSF3B1#2 Sense: GAACACCUAUAUUCGUUAU, Antisense: AUAACGAAUAUAGGUGUUC
- siSF3B1#3 Sense: CAGAGUUCCUGAACUGAAU, Antisense: AUUCAGUUCAGGAACUCUG
- siControl Sense: GCGCGCUUUGUAGGAUUCG, Antisense: CGAAUCCUACAAAGCGCGC

### 4.6. Transfection Protocol

Electroporation was used to transfect cells. Cells were transferred from T75 flasks (Corning) into sterile universal tubes and centrifuged at 150× *g* for 5 min at room temperature. Then, the supernatant was aspirated and cells were resuspended in order to have 1.5 × 10^7^ cells/mL in RPMI-1640 medium (Sigma-Aldrich, St. Louis, MO, USA) with 10% fetal calf serum and without antibiotics. 250 μL of cell suspension was added into the 4 mm electroporation cuvettes (VWR International) at a density of 1.5 × 10^7^ cells/mL. Then, the appropriate siRNA was added into each cuvette at 0.5 μM final concentration just before electroporation. After adding the siRNA, cuvettes were swirled gently to ensure that the cell suspension and siRNA was mixed evenly. Different electroporation parameters (voltage and number of pulses) were tested to optimize transfection conditions. The condition that resulted in the greatest cell viability and maximal siRNA knockdown (300 volts; 1 pulse; 10 ms) was used for subsequent gene knockdown analysis. Experiments were performed using an EPI 2500 gene pulser (Fisher, Heidelberg, Germany).

After transfecting with siRNA, cells were incubated in electroporation cuvettes for 15 min at room temperature, and resuspended in the culture medium without antibiotics. Then, the cells were seeded into a 6-well plate (Corning) at a density of 1 × 10^6^ cells/mL. Silencing assessment was performed 24 h after siRNA transfection by Western blot analysis. Post-transfection cell viability was assessed by trypan blue exclusion assay.

### 4.7. Immunoblotting

The 1 × 10^6^ cells/mL (5 × 10^6^ cells/mL for primary CLL cells) were seeded in 2 mL per well of a 6-well plate (Corning) and subjected to the corresponding manipulation (siRNA transfection and/or exposure to siremadlin or idasanutlin). Protein lysates were harvested using 2% SDS lysis buffer at 24 h, heated at 95 °C for 10 min, and sonicated. Protein concentration was measured using a Pierce™ BCA Protein Assay Kit (Thermo Fisher Scientific, Waltham, MA, USA). Primary antibodies against p53 (DO-7) (#M7001, Dako), p21^WAF1^ (Calbiochem), SF3B1 (Sigma), MDM2 (Ab-1) (#OP46, Merck Millipore), Actin (Sigma) and secondary goat anti-mouse/rabbit horseradish peroxidase-conjugated antibodies (Dako) were used. All antibodies were diluted in 5% (*w*/*v*) non-fat milk or BSA in TBS-tween20. Proteins were visualized using enhanced chemiluminescence reagents (GE Healthcare, Chicago, IL, USA).

### 4.8. Statistical Analysis

Data of the repeated experiments were presented as the mean ± standard error of the mean (SEM) unless otherwise stated. Statistical tests were carried out using GraphPad Prism 6 software and all *p*-values represent unpaired *t*-tests of at least three independent repeats unless otherwise stated. The chi-square test was used to measure statistical difference between the *SF3B1*^WT^ and *SF3B1*^MUT^ groups. The log-rank test was used for comparing patient survival times. The *p* values in Figure 3 show statistical difference between the median values for the groups calculated using the Mann–Whitney *U* test. Multivariate analysis was performed using IBM SPSS Statistics version 29.0. One-way ANOVA with post hoc Tukey HSD was used for univariate analysis. The Bonferroni correction was applied to reduce the chances of obtaining false-positive results (type I errors) when multivariate analysis was performed.

## 5. Conclusions

Our current study describes for the first time a comparison of *SF3B1* mutational status with ex vivo treatment response to the MDM2 inhibitor RG7388 in primary CLL samples. It is a potential therapeutically noteworthy finding that confirms *SF3B1* genetic status as a significant predictive biomarker for the potential use of MDM2 inhibitors, and perhaps may also be relevant for other targeted therapies. This could thus provide insights for current and prospective pre-clinical and clinical CLL research trials.

## Figures and Tables

**Table 1:** Clinical and molecular characteristics of patients with chronic lymphocytic leukemia.

Features		*SF3B1*^WT^ No. (%)	*SF3B1*^MUT^ No. (%)	^1^ *p* Value
AGE (years)	Median (range)	71 (32–92)	67.5 (45–86)	
OS (months)	Median (range)	214 (1–569)	90 (7–195)	*0.032*
TTFT (months)	Median (range)	140 (1–359)	66 (1–185)	0.094
SEX	Male	141 (66%)	17 (61%)	0.67
	Female	72 (34%)	11 (39%)	
BINET STAGE	A	101 (56%)	10 (50%)	0.22
	B-C	78 (44%)	10 (50%)	
	Unknown	34	8	
^2^ *IGVH* STATUS	Mutated	106 (61%)	10 (48%)	0.25
	Unmutated	68 (39%)	11 (52%)	
	Unknown	39	7	
CD38 EXPRESSION	CD38 negative (<30%)	78 (81%)	5 (36%)	*0.0004*
	CD38 positive (≥30%)	18 (19%)	9 (64%)	
	Unknown	117	14	
*TP53* STATUS	Mutated	30 (16%)	4 (15%)	0.93
	Unmutated	163 (84%)	22 (85%)	
	Unknown	20	2	
RECEIVED ANY TREATMENT	Yes	90 (44%)	12 (46%)	0.68
	No	115 (56%)	14 (54%)	
	Unknown	8	2	
^3^ FISH STRATIFICATION	Low risk	139 (69%)	16 (64%)	0.51
	Intermediate/high risk	62 (31%)	9 (36%)	
	Unknown	12	3	

^1^ The *p* values for the overall survival (OS) and time to first treatment (TTFT) were produced by the log-rank test, while the others were produced by Fisher’s exact test. ^2^ *IGVH*, immunoglobulin variable region heavy chain. ^3^ Fluorescent in situ hybridization (FISH) stratification: low risk, no abnormalities or isolated del13q; intermediate/high risk, del17p, del11q, trisomy 12 or complex karyotype (≥2 abnormalities).

**Table 2:** Univariate and multivariate analysis for response to RG7388.

Variable	No. (%)	RG7388 LC_50_ (µM)	Univariate			Multivariate		
			^1^ *η*^2^	95% CI	^2^ Sig.	^1^ *η_p_*^2^	95% CI	^3^ Sig.
*SF3B1* Status Wild type Mutant	65 (83) 13 (17)	≤1 vs. 1<…<10 ≤1 vs. ≥10		−0.29/0.19	0.863		−0.27/0.32	1
				−0.71/−0.06	0.018		−0.70/−0.12	**0.024**
		1<…<10 vs. ≤1 1<…<10 vs. ≥10	0.095	−0.19/0.29	0.863	0.078	−0.32/0.27	1
				−0.70/0.03	0.083		−0.77/0.13	0.257
		≥10 vs. ≤1 ≥10 vs. 1<…<10		0.06/0.71	0.018		0.12/0.70	**0.024**
				−0.03/0.70	0.083		−0.13/0.77	0.257
*TP53* Status Wild type Mutant	52 (83) 11 (17)	≤1 vs. 1<…<10 ≤1 vs. ≥10		−0.74/−0.24	<0.001		−0.74/−0.11	**0.005**
				−0.51/0.11	0.263		−0.57/0.32	1
		1<…<10 vs. ≤1 1<…<10 vs. ≥10	0.269	0.24/0.74	<0.001	0.214	0.11/0.74	**0.005**
				−0.07/0.65	0.139		−0.19/0.79	0.396
		≥10 vs. ≤1 ≥10 vs. 1<…<10		−0.11/0.51	0.263		−0.32/0.57	1
				−0.65/0.07	0.139		−0.79/0.19	0.396
^4^ *IGVH* Status Wild type Mutant	15 (36) 27 (64)	≤1 vs. 1<…<10 ≤1 vs. ≥10		−0.38/0.56	0.888			
				−0.14/0.91	0.193			
		1<…<10 vs. ≤1 1<…<10 vs. ≥10	0.075	−0.56/0.38	0.888			
				−0.34/0.92	0.502			
		≥10 vs. ≤1 ≥10 vs. 1<…<10		−0.91/0.14	0.193			
				−0.92/0.34	0.502			
Treatment Received Yes No	30 (42) 42 (58)	≤1 vs. 1<…<10 ≤1 vs. ≥10		−0.14/0.52	0.342			
				−0.41/0.55	0.941			
		1<…<10 vs. ≤1 1<…<10 vs. ≥10	0.028	−0.52/0.14	0.342			
				−0.66/0.40	0.834			
		≥10 vs. ≤1 ≥10 vs. 1<…<10		−0.55/0.41	0.941			
				−0.40/0.66	0.834			
CD38 Expression <30% ≥30%	28 (74) 10 (26)	≤1 vs. 1<…<10 ≤1 vs. ≥10		−0.22/0.58	0.516			
				−1.04/0.26	0.324			
		1<…<10 vs. ≤1 1<…<10 vs. ≥10	0.103	−0.58/0.22	0.516			
				−1.27/0.13	0.132			
		≥10 vs. ≤1 ≥10 vs. 1<…<10		−0.26/1.04	0.324			
				−0.13/1.27	0.132			
^5^ FISH Stratification Low risk Intermediate/ high risk	53 (76) 17 (24)	≤1 vs. 1<…<10 ≤1 vs. ≥10		−0.35/0.27	0.943			
				−0.56/0.34	0.833			
		1<…<10 vs. ≤1 1<…<10 vs. ≥10	0.006	−0.27/0.35	0.943			
				−0.57/0.44	0.947			
		≥10 vs. ≤1 ≥10 vs. 1<…<10		−0.34/0.56	0.833			
				−0.44/0.57	0.947			

^1^ Eta-squared is an effect size measure: it is a single, standardized number that expresses how different several subgroups means are. Generally accepted rules of thumb for eta-squared (*η*^2^) or partial eta-squared (*η*_p_^2^) are that *η*^2^ = 0.01 indicates a small effect; *η*^2^ = 0.06 indicates a medium effect; *η*^2^ = 0.14 indicates a large effect. ^2^ Tukey’s HSD post hoc test was applied for one-way ANOVA. ^3^ Adjustment for multiple comparisons: Bonferroni. ^4^ *IGVH*, immunoglobulin variable region heavy chain. ^5^ Fluorescent in situ hybridization (FISH) stratification: low risk, no abnormalities or isolated del13q; intermediate/high risk, del17p, del11q, trisomy 12 or complex karyotype (≥2 abnormalities). CI: confidence interval.

## Data Availability

The data presented in this study and released under a CC-BY 4.0 license are available upon request from the corresponding authors.

## Supplementary Materials

The following supporting information can be downloaded at: https://www.mdpi.com/article/10.3390/ijms241411335/s1.
