# Supplementary material for: SF3B1 Mutations Are Associated with Resistance to Non-Genotoxic MDM2 Inhibition in Chronic Lymphocytic Leukemia
Source: Int J Mol Sci. 2023 Jul 12;24(14):11335. doi: 10.3390/ijms241411335 (PMC10379212; doi:10.3390/ijms241411335)
Supplement: Supplementary file 1 [file ijms-24-11335-s001.zip › ijms-2437509-supplementary.pdf]

Supplementary Table S1. List of patients with CLL that carry *SF3B1* mutations.

**Clinical information**				**p53**		***SF3B1* mutation**		
**[1]Tumour ID**	**Age**	**Sex**	**Cytogenetic abnormalities**	**[2]*TP53* status**	**LC_{50} RG7388 (μM)**	**[3]Clone size (%)**	**[4]p_description**	**c_description**
**27**	79	M	del(13q) del(11q)	WT		50	p.K700E	c.2098A>G
**49**	64	M	del(17p)	p.D208V (73%)		50	p.K700E	c.2098A>G
**63**	61	F	del(13q) del(11q) del(17p) 17%	WT		40	p.K700E	c.2098A>G
**64**	74	M	del(11q)	WT		50	p.K741N	c.2223G>T
**81 (158,233)**	60	M	del(13q) del(11q)	WT		30	p.K700E	c.2098A>G
**88**	60	M	None	WT		30	p.K741N	c.2223G>T
**89(193)**	60	M	del(13q)	WT		10	p.K700E	c.2098A>G
**105**	66	F	None	WT		50	p.G742D	c.2225G>A
**158 (81,233)**	61	M	del(13q) del(11q)	WT		50	p.K700E	c.2098A>G
**169**	57	M	del(13q)	WT		20; 30	p.K700E ; p.R775G	c.2098A>G ; c.2323C>G
**183**	67	F	None	WT		50	p.K666E	c.1996A>G
**188**	68	M	None	WT		30	p.G742D	c.2225G>A
**193 (89)**	63	M	del(13q)	WT		20	p.K700E	c.2098A>G
**199**	45	M	del(13q) del(17p)	c.375+2T>G p.?, Splice (39%)		50	p.K666T	c.1997A>C
**207**	81	F	del(13q)	WT		50	p.K700E	c.2098A>G
**221**	86	M	None	WT	0.17	50	p.W658C	c.1974G>T
**225 (272)**	66	F	None	WT	0.2	40	p.K700E	c.2098A>G
**233 (81,158)**	66	M	del(13q) del(11q)	WT	>10	50	p.K700E	c.2098A>G
**240**	72	F	None	WT	>10	20	p.K666E	c.1996A>G
**243**	76	F	None	WT	0.72	50	p.K700E	c.2098A>G
**270**	75	M	del(13q)	WT	0.35	50	p.Y623C	c.1868A>G
**272 (225)**	69	F	None	WT	0.33	50	p.K700E	c.2098A>G
**283**	69	F	None	p.R249G (48%)	9.2	30	p.G742D	c.2225G>A
**287**	69	M	del(13q) del(11q)	p.R175H (50%)	2.7	30	p.K700E	c.2098A>G
**288**	72	F	None	WT	>10	50	p.G740E	c.2219G>A
**290**	65	M	None	WT	>10	50	p.I704F	c.2110A>T
**299**	71	F	None		6	50	p.R775Q	c.2324G>A
**305**	81	M	None		0.3	50	p.K741N	c.2223G>C

[1]Numbers in brackets show the sequential samples from the same patient (e.g. 81(158, 233)).
[2]NGS was used to assess the mutational status of *TP53* (WT:wild-type, or mutation specified with mutant allele frequency in brackets).
[3]Sanger sequencing was used to detect *SF3B1* mutations. Approximate mutant clone percentages obtained from Sanger sequencing chromatograms are shown.
[4]Amino acid positions of the frequently mutated coding sites: 603-693 (exon14), 693-741 (exon15), 742-790 (exon16).
Blank boxes indicate that there is no data available.

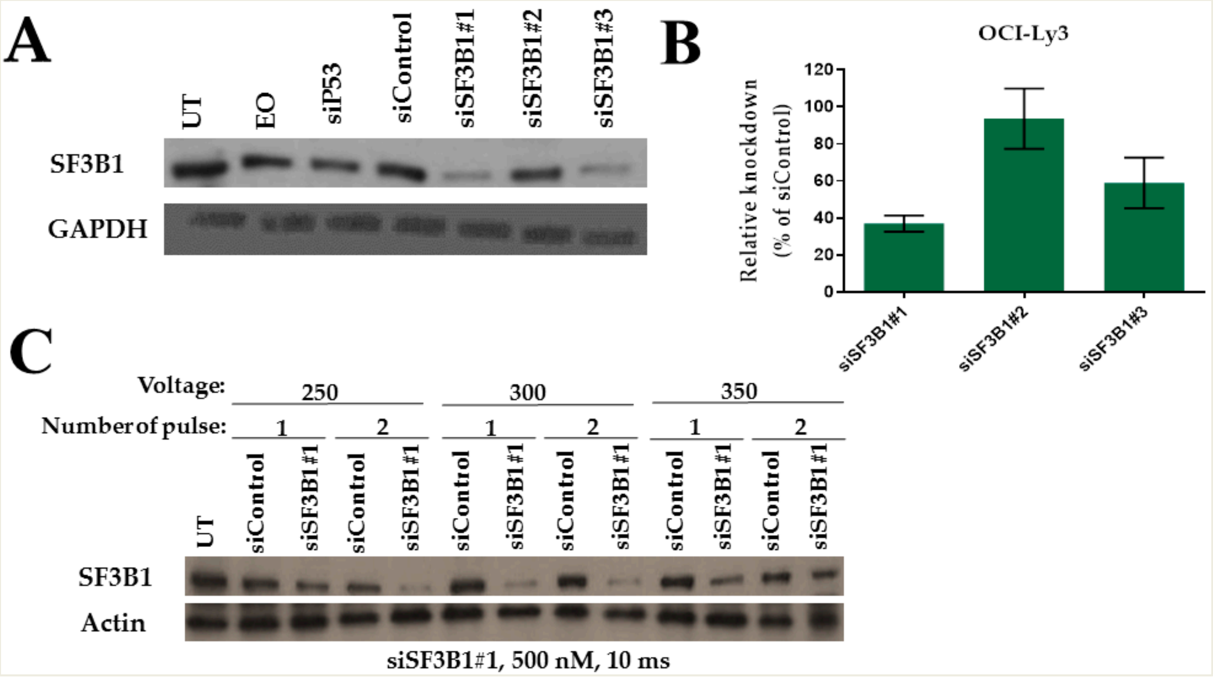

Supplementary Figure S1. Optimization for siRNA delivery in OCI-Ly3 cells. (A) Silencing of SF3B1 using three different siRNA constructs (siSF3B1#1, siSF3B1#2, and siSF3B1#3) in OCI-Ly3 cells under the same electroporation conditions (500 nM siRNA; 300 volts; 1 pulse; 10 ms). Representative western blot shows the SF3B1 protein levels 24 hours after siRNA transfection. GAPDH was used as loading control. UT: untreated; EC: electroporation only control; siP53: control targeting different mRNA **(B)** SF3B1 protein levels were normalized to siControl and loading control for the three different siSF3B1 constructs. ImageJ 1.41 image processing was used to make quantitative measurement of detected protein bands of interest. Error bars show the mean±SEM for three independent repeats. **(C)** Knockdown of SF3B1 by 500 nM siSF3B1#1 was assessed for varied electroporation conditions including three different voltages (250, 300, or 350 volts) and two different number of pulses (1 or 2 pulse/s) by western blotting. Actin was used as loading control.
